# 2020 update on the clinical validity of cerebrospinal fluid amyloid, tau, and phospho-tau as biomarkers for Alzheimer’s disease in the context of a structured 5-phase development framework

**DOI:** 10.1007/s00259-021-05258-7

**Published:** 2021-03-05

**Authors:** A. Leuzy, N. J. Ashton, N. Mattsson-Carlgren, A. Dodich, M. Boccardi, J. Corre, A. Drzezga, A. Nordberg, R. Ossenkoppele, H. Zetterberg, K. Blennow, G. B. Frisoni, V. Garibotto, O. Hansson

**Affiliations:** 1 Clinical Memory Research Unit, Department of Clinical Sciences, Lund University, SE-205 02 Malmö, Sweden; 2 Department of Psychiatry and Neurochemistry, The Sahlgrenska Academy, University of Gothenburg, Mölndal, Sweden; 3 Department of Old Age Psychiatry, Institute of Psychiatry, Psychology & Neuroscience, King’s College London, London, UK; 4 Wallenberg Centre for Molecular and Translational Medicine, University of Gothenburg, Gothenburg, Sweden; 5 Department of Neurology, Skåne University Hospital, Lund, Sweden; 6 Wallenberg Centre for Molecular Medicine, Lund University, Lund, Sweden; 7 NIMTlab—Neuroimaging and Innovative Molecular Tracers Laboratory, University of Geneva, Geneva, Switzerland; 8 Center for Neurocognitive Rehabilitation (CeRiN), CIMeC, University of Trento, Trento, Italy; 9 German Center for Neurodegenerative Diseases (DZNE), Rostock, Germany; 10 LANVIE—Laboratory of Neuroimaging of Aging, University of Geneva, Geneva, Switzerland; 11 Centre National de la Recherche Scientifique, Montpellier, France; 12 Medical Faculty, University Hospital of Cologne, Cologne, Germany; 13 Division of Clinical Geriatrics, Center for Alzheimer Research, Department of Neurobiology, Care Sciences and Society, Karolinska Institutet, Stockholm, Sweden; 14 Theme Aging, Karolinska University Hospital, Stockholm, Sweden; 15 Alzheimer Center Amsterdam, Department of Neurology, Amsterdam Neuroscience, Vrije Universiteit Amsterdam, Amsterdam UMC, Amsterdam, The Netherlands; 16 Clinical Neurochemistry Laboratory, Sahlgrenska University Hospital, Gothenburg, Sweden; 17 Department of Neurodegenerative Disease, UCL Queen Square Institute of Neurology, London, UK; 18 UK Dementia Research Institute, UCL, London, UK; 19 Memory Clinic, University Hospital, Geneva, Switzerland; 20 Memory Clinic, Skåne University Hospital, SE-205 02 Malmö, Sweden

**Keywords:** Alzheimer’s disease, CSF, strategic roadmap, Aβ42, P-tau, T-tau

## Abstract

**Purpose:**

In the last decade, the research community has focused on defining reliable biomarkers for the early detection of Alzheimer’s disease (AD) pathology. In 2017, the Geneva AD Biomarker Roadmap Initiative adapted a framework for the systematic validation of oncological biomarkers to cerebrospinal fluid (CSF) AD biomarkers—encompassing the 42 amino-acid isoform of amyloid-β (Aβ42), phosphorylated-tau (P-tau), and Total-tau (T-tau)—with the aim to accelerate their development and clinical implementation. The aim of this work is to update the current validation status of CSF AD biomarkers based on the Biomarker Roadmap methodology.

**Methods:**

A panel of experts in AD biomarkers convened in November 2019 at a 2-day workshop in Geneva. The level of maturity (fully achieved, partly achieved, preliminary evidence, not achieved, unsuccessful) of CSF AD biomarkers was assessed based on the Biomarker Roadmap methodology before the meeting and presented and discussed during the workshop.

**Results:**

By comparison to the previous 2017 Geneva Roadmap meeting, the primary advances in CSF AD biomarkers have been in the area of a unified protocol for CSF sampling, handling and storage, the introduction of certified reference methods and materials for Aβ42, and the introduction of fully automated assays. Additional advances have occurred in the form of defining thresholds for biomarker positivity and assessing the impact of covariates on their discriminatory ability.

**Conclusions:**

Though much has been achieved for phases one through three, much work remains in phases four (real world performance) and five (assessment of impact/cost). To a large degree, this will depend on the availability of disease-modifying treatments for AD, given these will make accurate and generally available diagnostic tools key to initiate therapy.

**Supplementary Information:**

The online version contains supplementary material available at 10.1007/s00259-021-05258-7.

## Introductions

In 2017, a methodological framework for the systematic assessment of biomarker validation was imported from oncology [1] and adapted to Alzheimer’s disease (AD) [2]. Within this “Biomarker Roadmap” initiative, the validation status of well-consolidated biomarkers at that time [3] was assessed in the context of their use in clinical practice in patients presenting to memory clinics with mild cognitive impairment (MCI). Biomarkers included episodic memory [4], medial temporal atrophy [5], [^18^F]fluoro-deoxyglucose ([^18^F]FDG) positron emission tomography (PET) [6], Amyloid PET [7], ^123^I-ioflupane brain single photon emission tomography, and ^123^I-MIBG cardiac scintigraphy [8]. Cerebrospinal fluid (CSF)-based biomarkers for AD—low levels of the 42-amino acid form of Aβ (Aβ42) and elevated levels of phosphorylated and total tau (P-tau and T-tau, respectively)—were also reviewed (here collectively referred to as “CSF AD biomarkers”) [9].

According to the previous review on CSF AD biomarkers [9], based on the evidence until 2015, these measures showed partial achievement of analytical and clinical validity, with large prospective real-world studies ongoing at that time. Since then, the field of CSF biomarkers has evolved significantly, especially through the introduction and extensive deployment of certified reference methods and materials for Aβ42 and fully automated assays. The aim of this work is to update the current validation status of CSF AD biomarkers based on the Biomarker Roadmap methodology.

## Methods

### Target

This literature review investigates the validation status of CSF Aβ42, P-tau and T-tau as AD biomarkers, in accordance with the 2020 update (Boccardi et al., in this issue) of the Biomarker Roadmap [2, 3]. The target population consists of patients with MCI referring to memory clinics due to cognitive complaints, attributed to possible sporadic and not familial dementing neurodegenerative disorders. Validation studies of CSF biomarkers were eligible for this review when including AD neuropathology, in vivo detection of Aβ deposition, or development of incidental AD dementia after at least 2 years of follow-up as reference standard for the biomarker-based diagnosis. Thus, eligible studies included both prospective longitudinal and cross-sectional studies.

### Glossary

#### Alzheimer’s disease

By AD, we mean the presence of extracellular Aβ plaques and aggregates of hyper-phosphorylated tau in neurofibrillary tangles. These features—which typically are associated with a pattern of mediotemporal and temporoparietal neurodegeneration—define AD independently of the clinical expression of cognitive symptoms [10].

#### Alzheimer’s disease dementia

AD dementia denotes an acquired and progressive deterioration in memory and other cognitive functions severe enough to lead to functional impairment in activities in everyday life, according to previous criteria as defined by the National Institute of Neurological and Communicative Disorders and Stroke and the Alzheimer’s disease and Related Disorders Association (NINCDS-ADRDA) criteria [11]. Notably, because of the imperfect accuracy of purely clinical criteria, a percentage of AD dementia cases will have non-AD pathology, or mixed AD and other types of pathology [12–14].

#### Mild cognitive impairment

This refers to individuals without, or with subtle, functional disability (i.e., no dementia), but with an acquired objective cognitive impairment. Representing a clinical syndrome, it encompasses cases progressing to AD (about 40–60%) or non-AD dementia (about 10%-30%; [15–17]) as well cases who are stable during several years (about 30–50%). MCI cases positive for AD biomarkers can be defined as prodromal AD based on research diagnostic criteria [18, 19]. The diagnosis of AD at the MCI stage represents the focus of the present review.

#### Non-Alzheimer’s disease neurodegenerative disorder

This term refers to all neurodegenerative disorders considered in the context of differential diagnosis, including progressive supranuclear palsy, corticobasal degeneration, non-fluent primary progressive aphasia, Parkinson’s disease with dementia and subcortical vascular dementia.

The term is considered independent of the clinical manifestations of these diseases.

### Conceptual framework

As described above, the conceptual framework for this review stems from the field of oncology [1], and has been described in detail by Boccardi et al. [2] and updated in 2020 (Boccardi et al, in this issue). Here, we summarize the application of this methodological framework to the use of CSF AD biomarkers for diagnostic purposes in routine clinical settings. Specifically, all aims are qualified as “fully achieved,” “partly achieved,” “preliminary evidence,” or “not achieved” based on the available evidence.

#### Phase 1

This phase includes preclinical exploratory studies on the rationale for using CSF Aβ42, P-tau, and T-tau for diagnostic purposes in AD and was already fully achieved in 2017. As a result, this phase will not be covered in the present work.

#### Phase 2

Phase 2 studies investigate the diagnostic accuracy of CSF AD biomarkers to distinguish patients with AD dementia from cognitively unimpaired (CU) controls and subjects with non-AD dementia disorders. Phase 2 studies are meant to define the clinical assay to allow reliable assessment and identify the effect of confounders on the threshold for positivity in both patients and controls (e.g., age, gender, apolipoprotein ε4 (*APOE* ε4) status, education or comorbidities). As the primary (discriminative accuracy between subjects with and without the disease) and second secondary aims (relationship between CSF measures and neuropathology) were deemed to be fully achieved in 2017, these have been omitted from the present work.

#### Phase 3

Phase 3 studies assess the ability of CSF AD biomarkers to detect AD pathology early on in the disease course (namely MCI for this specific effort) in well-controlled experimental samples. Phase 3 studies aim to define criteria for positivity, to compare the diagnostic performance with other biomarkers, and to assess the diagnostic value of combinations of biomarkers with a view to defining biomarker-based algorithms. As the primary aim of phase 3 (capacity of the biomarker to detect AD in subjects with MCI, using conversion to AD-dementia as the standard of truth—SOT) was assessed as fully achieved it 2017, it will not be covered in the present work.

#### Phase 4

Phase 4 studies assess the performance of CSF AD biomarkers in representative patient cohorts from memory clinics. The biomarker itself is used to support a clinical diagnosis in patients with MCI who are subsequently treated based on this CSF-supported diagnosis. They are meant to quantify the benefit of CSF-based early detection, as well as their practical feasibility and associated protocol compliance. Preliminary evidence about costs is an additional aim, in view of dedicated studies in Phase 5.

#### Phase 5

Phase 5 studies evaluate the impact of diagnosis based on CSF biomarkers on society (e.g., cost-effectiveness relative to clinically meaningful outcomes).

### Evidence assessment

The fulfillment of each validation step from Phase 2 through 5 was assessed consistent with the approach used in the earlier 2017 Biomarker Roadmap (Boccardi et al., 2018). As such, primary and secondary aims for each phase were rated as follows: fully achieved, partly achieve, preliminary evidence, not achieved, or unsuccessful, as defined below. To facilitate the assessment and make it transparent to the readers, the data used to define the degree of fulfilment for each aim are reported and summarized in tables accessible online (see Online Resource at https://nextcloud.dzne.de/index.php/s/so3ACxTH9n3yzdq). Adapted from a previous effort specific to AD and related disorders (Boccardi et al., 2018), these tables can facilitate formal evidence assessment [20, 21].

#### Fully achieved

Available scientific evidence successfully replicated in properly powered and well-designed studies.

Methodologically sound and well-powered studies have provided convincing evidence that has been replicated.

#### Partly achieved

The available evidence is not sufficiently replicated, or samples are not adequately powered, or studies have major methodological limitations.

#### Preliminary evidence

Only preliminary evidence is available.

#### Not achieved

Studies are not yet performed at the time of the review.

#### Unsuccessful

Available scientific evidence shows a failure for the biomarker in achieving the aim. Findings in the subsequent roadmap phases should be interpreted with caution.

### Search for and selection of papers

Phase- and aim-specific PubMed search strings are provided in Online Resource 1.

## Results

**Fig. 1:**
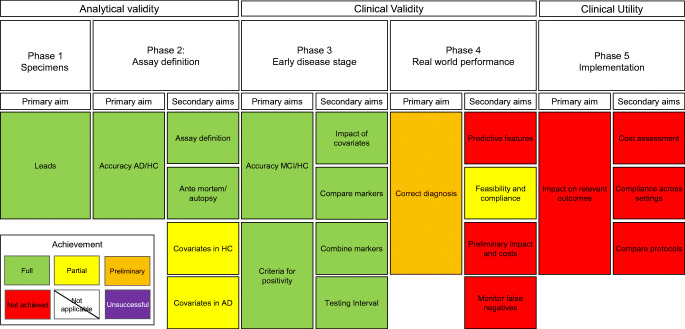
A flowchart illustrating the development of CSF biomarkers for AD in the framework of Pepe et al. (2001) [1]. Abbreviations: AD, Alzheimer’s disease; CSF, cerebrospinal fluid; HC, healthy controls; MCI, mild cognitive impairment

Figure 1 provides an overview of the current state of CSF AD biomarkers, as per our methodological framework [1].

### Phase 2: Clinical assay development for AD pathology

The overarching aim of phase 2 studies is to characterize the ability of CSF AD biomarkers to separate patients with AD dementia from both CU controls and subjects with non-AD dementia disorders.

#### Phase 2: Secondary aim 1

The first secondary aim of phase 2 addresses optimization of the operating procedures and assessment of the reproducibility of the assay within and between laboratories. The secondary aim 1 of phase 2 is now fully achieved for CSF Aβ42, for which certified reference methods and materials for assay standardization are in place [22]. Similar work is ongoing but has not yet been completed for CSF tau biomarkers.

Concentrations of CSF AD biomarkers can be affected by operator-influenced preanalytical variables [23, 24], including sampling materials and methodology as well as handling and storage procedures [23, 25–32]. The consensus within the field is that together with appropriate use criteria [33], certified reference methods and materials [22], and high precision measurements [34], the standardization of these factors will reduce variability and increase the diagnostic accuracy of these measures; this, in turn, will facilitate widespread use of CSF AD biomarkers in both clinical research and routine clinical practice [27, 35]. Recent work addressing the influence of pre-analytical factors on both CSF Aβ42 and Aβ40 has provided an initial protocol covering temperature and storage time, centrifugation, sample mixing, and blood contamination [35].

The most commonly used technique to measure CSF AD biomarkers has been the enzyme-linked immunosorbent assay (ELISA) using commercial assays [36–38]. Though reproducibility has been shown to be achievable for these measures when running analyses according to strict standard operating procedures in a single accredited laboratory [39], variability has been reported across studies [40, 41] due to preanalytical (i.e., specimen collection, shipment/storage), analytical (i.e., procedures between laboratories) and assay-related (i.e., immunoassay manufacturing procedures) factors [42]. Though a quality control (QC) program was launched by the Alzheimer’s Association in 2009 in an attempt to address this, including the introduction of standard operating procedures for the ELISA methods [43], between laboratory measurement variability for CSF AD biomarkers has been consistently reported as between 15 and 25% [44]. This persistent variability has hampered the introduction of universal biomarker cut-off values and made clear the need for more precise automated techniques.

The first publication describing the full validation and analytical performance of such an automated approach was in 2016 [45]. Using a fully automated electrochemiluminescence immunoassay (Cobas Elecsys®) for CSF Aβ42, Bittner and colleagues reported repeatability coefficients of variation (CV) for human CSF pools of 1.0 to 1.6% and intermediate CVs of 1.9 to 4.0%. Moreover, the assay showed very low variability between lots due to its standardisation against candidate reference materials for which the absolute concentration of Aβ42 was measured using a now formally certified reference method [46]. The Elecsys® assay has now been a part of the Alzheimer’s Association QC program since 2014, with mean CVs being as low as 4% as compared to ~15% for ELISA methods [47]. Novel assays on the Elecsys® instrument for P-tau and T-tau have also recently been described [26, 48, 49] and have so far performed well in the Alzheimer’s Association QC program [44]. Similar automated platforms for AD biomarkers have since been launched, including those from Euroimmun [50], and Fujirebio (LUMIPULSE®) [51–54], and have shown superior performance in the QC program [44]. The certified reference materials for Aβ42 have now been fully implemented and will allow for full standardization of all commercially available CSF Aβ42 methods [22]. Work to develop certified reference methods and materials for T-tau and P-tau is ongoing, under the auspices of the International Federation of Clinical Chemistry and Laboratory Medicine and the Global Biomarker Standardization Consortium.

#### Phase 2: Secondary aim 3

To assess covariates (such as gender, age, etc.) associated with biomarker status or level in control subjects. If there is an effect on the biomarker, define thresholds for positivity in each concerned subpopulation. The secondary aim 3 of phase 2 remains partially achieved.

The effect of demographic factors, such as age, sex and *APOE* ε4 status, on CSF AD biomarkers in CU individuals has been the focus of several studies. In one such study, T-tau but not Aβ42, has been shown to correlate positively with age in CU individuals [55]. In a related study, P-tau and T-tau, but not Aβ42 were also found to positively correlate with age in CU individuals [56]; as a result, specificities and negative predictive values in controls were found to decrease for CSF tau measures with advancing age, likely as a result of an increase in the prevalence of Aβ positivity. Here, however, while the authors stressed the importance of careful characterization of control cohorts when including elderly CU individuals, they did not suggest the use of age-adjusted cutoffs for tau. Additional studies, however, have found age to be positively associated with T-tau only [57] or both tau (P-tau and T-tau) and Aβ42 [58, 59], though only weakly for Aβ42.

The *APOE* ε4 allele has been shown to be strongly linked to reduced levels of CSF Aβ42 in controls [59–63]. However, CSF levels of Aβ42 are not associated with APOE ε4 when accounting for cortical Aβ pathology (as indexed by Amyloid PET), indicating a link between CSF Aβ42 and cortical Aβ levels that is *APOE* ε4-independent, i.e., low CSF Aβ42 reflects brain amyloidosis independently of *APOE* genotype [64, 65]. As a result, it has been suggested that CSF Aβ42 cutoffs should not be adjusted for *APOE* genotype [64]**.** Interestingly, *APOE* ε4 has also been shown to interact with age and gender [66], such that in ε4 non-carriers, CSF Aβ42 levels followed a monotonic trajectory over time for women, with men showing an increase up to midlife followed by a levelling off. Among *APOE* ε4 carriers, males showed a modest decline in CSF Aβ42 over time, while women showed a sharper decline in Aβ42, starting at around age 50 and coinciding with the onset of menopause. *APOE* ε4 has also shown a stronger association to CSF P-tau and T-tau levels in Aβ-positive women compared to Aβ-positive men [67]; this finding, combined with *APOE* ε4 not being differentially associated with neurofibrillary tangles at post-mortem, suggests that in the presence of Aβ pathology, *APOE* may regulate the risk for neurodegeneration in a sex-specific fashion [68, 69]. Age- and gender-specific cutoffs have not been proposed for CSF Aβ42, however.

#### Phase 2: Secondary aim 4

To assess covariates (e.g., gender, age, etc.) associated with biomarker status or level in diseased subjects. The secondary aim 4 of phase 2 remains partially achieved.

Older age, female sex, and *APOE* ε4 carriership are associated with AD [70]; as such, CSF AD-related biomarkers are more common in these groups. In patients with MCI, *APOE* ε4 is associated with both reduced Aβ42 [64, 71] and increased tau levels [60, 71, 72]. In *APOE* ε4 carriers, age is associated with lower Aβ42 and higher P-tau levels [73] while female *APOE* ε4 carriers show a more AD-like CSF profile compared to men [74, 75]. Levels of CSF Aβ42 had also been found to be significantly lower in *APOE* ε4 non-carriers and carriers with one ε4 allele who were aged 65 and over. Age-dependent increases were not observed for P-tau or T-tau; however [76]; Mofrad et al. [77] found that in female *APOE* ε4 carriers, higher levels of CSF P-tau and T-tau were seen at the SCD and MCI stage; these differences were not seen, however, when looking at those with AD dementia. Among female MCI patients who were *APOE* ε4 non-carriers, higher P-tau and T-tau levels were seen in MCI and AD dementia, but not in SCD. No sex differences in Aβ42 concentrations were found between females and males for any disease stage or *APOE* genotype. Higher levels of CSF Aβ42, P-tau and T-tau have also been reported in Aβ-positive MCI who are *APOE* ε4-negative, as compared to Aβ-positive MCI who are *APOE* ε4-positive [78]. Despite these differences, however, there is as yet no evidence showing that CSF AD biomarkers are less predictive of AD pathology in any of these groups.

### Phase 3: Longitudinal repository studies

The general aim of phase 3 studies is to define the ability of the biomarker to detect the disease in its early phase. For this initiative, we have chosen to focus on MCI. This phase is now largely fully achieved.

#### Phase 3: Primary aim 2

To define criteria for a positive biomarker test in preparation for phase 4. The phase 3 primary aim 2 is now fully achieved for CSF Aβ42, and partially achieved for P-tau and T-tau.

A variety of statistical approaches have been proposed to dichotomize continuous CSF AD biomarkers as normal or abnormal [79]. Common approaches include the maximization of accuracy for clinically diagnosed AD dementia or choosing a cut-point that yields a predefined level of sensitivity or specificity [80, 81]. In clinical chemistry, biomarker cut-offs are commonly defined as the 95% confidence interval in people without disease. Complicating these approaches, however, is the fact that pathological brain changes can be seen prior to CSF AD biomarkers having become clearly abnormal [82] and clinically silent AD pathology in CU individuals [56]; these scenarios can lower the sensitivity and specificity of CSF AD biomarkers, respectively, at the MCI stage. Another approach, Gaussian mixture modelling, involves the use of an expectation maximization algorithm cluster individuals according to their probability of belonging to a given group (e.g., normal and abnormal CSF Aβ42) [83]. While suitable for CSF Aβ42 due its bimodal distribution this approach is less well suited to CSF tau measures due their having a more unimodal distribution. Autopsy-confirmed AD dementia cases [84] and Amyloid PET [85] have also been used to define cutoffs; both approaches, however, are not bias free (e.g., delay between CSF sampling and death, lack of CSF sample-tissue matchings from healthy controls; for PET; differences in how the data is acquired and processed and how Aβ positivity thresholds are calculated [7].

Thus far, three studies have examined cutoffs for CSF Aβ42 and ratios with tau measurements derived using fully automated Elecsys® immunoassays [26, 86, 87]. In the study by Hansson et al. [26], cut-offs of 1100 pg/mL (Aβ42), 0.022 (P-tau/Aβ42) and 0.260 (T-tau/Aβ42) were established based on concordance between CSF biomarkers and Amyloid PET in BioFINDER. When applying these predefined cutoffs to ADNI, a high concordance was observed between CSF and Amyloid PET classifications (overall percent agreement 89–90%; AUC 96%). Using three approaches to generate cut-offs—comparison to AlzBio3, mixture modeling and concordance with Amyloid PET—Shaw et al. [86] likewise arrived at 1100 pg/mL as a cut-off for Elecsys® Aβ42; similar cut-offs to those reported by Hanson et al. were also found for P-tau/Aβ42 (0.023) and T-tau/Aβ42 ratios (0.289). Similar cut-offs (based on Youden index for separating participants based on Aβ-status using Amyloid PET) were also reported by Schindler et al. [87, 88] (CSF Aβ42, 1098 pg/mL; P-tau/Aβ42, 0.0198; T-tau/Aβ42, 0.211). In addition, the LUMIPULSE® assay, that provides a quantitative result for an analyte within 35 min, demonstrates an Aβ42/Aβ40 cut-off of 0.068 for an AD diagnoses but is also validated against Amyloid PET [89]. These methods are now being standardized to each other in terms of the absolute CSF Aβ42 concentration they deliver [22].

#### Phase 3: Secondary aim 1

The secondary aim 1 of phase 3 is to explore the impact of covariates on the discriminatory abilities of the biomarker at the MCI stage. This aim is now fully achieved.

Though the specificities of individual CSF AD biomarkers have been found to decrease with age [90, 91], the specificity of the three markers combined (for separating stable MCI from prodromal AD) has been shown to remain essentially stable with increasing age [56]. As such, while the authors stressed the importance of careful cut-off selection, they concluded that age-adjusted cutoffs were not necessary. In a further study, motivated by findings that the diagnostic ability of Aβ42 could be adversely affected by the *APOE* ε4 allele [9, 60], Lautner et al. examined the association between *APOE* genotypes and levels of CSF Aβ42, T-tau and P-tau in MCI cases that were followed longitudinally for at least 2 years [64]. The authors found that while *APOE* ε4 was associated with lower levels of CSF Aβ42, the diagnostic performance of the biomarker was independent on *APOE* genotype. On this basis, they concluded that the CSF Aβ42 cut-offs should not be adjusted according to *APOE* genotype.

#### Phase 3: Secondary aim 2

To compare the different biomarkers available in order to select the most promising ones. The second secondary aim of phase 3 is fully achieved.

A decrease in Aβ42 and shorter Aβ isoforms (i.e., Aβ40 and Aβ38) can be seen in non-AD dementia disorders characterized by subcortical changes (e.g., frontotemporal dementia, vascular dementia and dementia with Lewy bodies) [92–95], likely due a decline in overall Aβ production levels [96] and/or neuronal activity levels [97, 98]. The use Aβ42/Aβ40 or Aβ42/Aβ38 ratios has been shown to increase accuracy compared to Aβ42 alone for distinguishing AD (true Aβ42-positive) from such conditions, where false positives can arise if only using Aβ42 [95, 99].

Generally, T-tau is increased in patients with MCI who progress to AD dementia within a time frame of 5 years [100]. While the accuracy of P-tau is, by comparison, somewhat lower, with respect to the detection of prodromal AD, it remains an important marker as high CSF P-tau levels are generally not found in non-AD neurodegenerative disorders [101]. Several studies, however, have shown that the combination of T-tau or P-tau with Aβ42 increases the predictive power for AD at the MCI stage [100, 102–108]. As the ratio of Aβ42 to T-tau can be artificially increased via increases in T-tau levels only, however, (e.g., due acute brain disorders such as trauma or stroke) it has been recommended that CSF AD biomarkers are to be interpreted as independent measures [9]. When applying the Aβ (A), tau (T) and neurodegeneration (N) (A/T/N) classification system using CSF AD biomarkers to extended follow-up data (up to 10 years), while the highest proportion of progression from MCI to AD dementia was seen in patients who were A+T+N+, progression was also common in patients showing A-T+N+ and A-T-N- [109].

Several studies that have compared CSF AD biomarkers with amyloid and Tau PET. Concordance between CSF Aβ42 and amyloid PET has been shown to be high (~90%) [110, 111]; the finding that discordance is mainly seen in the form of isolated CSF Aβ-positivity is likely due to CSF Aβ42 being a more sensitive marker of early Aβ pathology [112]. Similar findings have been described for CSF tau [36, 113–122], including recent longitudinal work showing that CSF P-tau clearly increases before Tau PET positivity [123]. Overall, these studies suggest that CSF Aβ42 and P-tau reflect the intensity of the AD process (stage markers) with amyloid and Tau PET, by contrast, reflecting how the density and distribution of AD pathology [124, 125] (i.e., how far the disease has progressed; stage marker) [114]. Only one study has to date [126], however, has examined the association between AD biomarkers and Tau PET using novel tau tracers now entering the field [127]; this study showed that while Tau PET using [^18^F]RO948 outperformed CSF AD biomarkers (Aβ42/Aβ40 and Aβ42/P-tau) for separating AD dementia from non-AD disorders, the reverse was seen when differentiating Aβ-positive MCI from non-AD disorders [126].

Though multiple phosphorylation sites exist on the tau protein [128], the most commonly used assays for P-tau use antibodies targeting phosphorylation at threonine 181 (P-tau181) or 231 (P-tau231) [38, 129]. Though P-tau181 and 231 are strongly correlated and exhibit similar diagnostic performance [130], P-tau231 may have greater sensitivity for NFTs as it been shown to detect tau pathology in layer II of the entorhinal cortex, an area considered to be the earliest site affected by tangles in AD [131]. Increasing evidence indicates the presence of tau fragments spanning both the mid-domain and various terminal regions [132, 133]. Though one such fragment, a C-terminally truncated ending at amino acid 368 (called Tau 368) was shown to be less altered in AD (including in Aβ-positive CU) another study however showed that the tau-368/T-tau ratio correlated with Tau PET [123]. This may reflect the deposition of Tau 368 into tangles and leaving less to be secreted to the CSF as compared to T-tau [134]. This would be in analogy with the lowering of the Aβ42/40 ratio in patients with brain amyloidosis. In a recent study [135], tau phosphorylated at threonine 217 (P-tau217) was shown to better correlate with [^18^F]flortaucipir, as compared to P-tau181, and to more accurately identify Tau PET-positive individuals. P-tau217 also better correlated with Amyloid PET and CSF Aβ42 and better differentiated AD dementia from non-AD disorders. Mass spectrometry-based measurements of the different tau phospho-forms corroborate these findings [136].

#### Phase 3: Secondary aim 3

Develop algorithms for the biomarker-based diagnosis of MCI in preparation of Phase-4. The third secondary aim of phase 3 is now fully achieved.

Several studies have explored whether the detection of prodromal AD can be improved by combining CSF AD biomarkers with cognitive tests and neuroimaging. CSF findings combined with MRI-based atrophy ratings have been shown to result in higher predictive power as compared to individual biomarkers [137, 138], with similar findings in studies that have grouped CSF with [^18^F]fluorodeoxyglucose ([^18^F]FDG) PET [139–141], and with MRI and [^18^F]FDG PET [142–144]. Other work has shown that while the Alzheimer’s Disease Assessment Score with 13 items showed the highest effect size for differentiating stable MCI from those who progressed to AD dementia [145], risk stratification was improved by the addition of CSF P-tau. In another study, combining hippocampal volume, Functional Activities Questionnaire (FAQ) scores, and a summary measure for memory with *APOE* e4 status and CSF T-tau/Aβ42 ratio best predicted conversion to AD dementia from MCI over a 4-year period [146]. Similar findings were also reported by Jang et al. [147]. Other studies have reported improved predictive performance for identifying incipient AD by combining CSF AD biomarkers with neuropsychological measures [148, 149].

Two studies by van Maurik et al. addressed individualized biomarker-based risk predictions of dementia in MCI patients [150, 151]. In a first proof-of-principal study [151], prognostic models providing probabilities of progression to AD dementia over the course of 1 year or 3 years were constructed based on a single-center cohort. The model combining MRI volumetric measures (hippocampal and whole-brain) and CSF (Aβ42 and T-tau) was found to provide the best prognostic value. In a follow-up multicentric study to establish the generalizability of this approach, van Maurik et al. [150] tested four separate prognostic models—including age, sex, CSF Aβ42, T-tau and MMSE, as well as a model combining A/T/N biomarkers using CSF Aβ42, P-tau181 and hippocampal volume. Though all models performed well, the highest performance was seen using the A/T/N based model.

#### Phase 3: Secondary aim 4

The secondary aim 4 of phase 3 is to determine a biomarker testing interval for phase 4 if repeated testing is of interest. Overall, there is no evidence supporting that repeated CSF measurements are needed when it comes to prediction of AD dementia in MCI, as the levels of the CSF AD biomarkers seem to be stable at this stage of the disease. This aim is fully achieved.

Several cross-sectional and longitudinal studies have addressed the dynamics of CSF AD biomarkers. Using data from the Dominantly Inherited Alzheimer Network, changes in CSF Aβ42 have been shown to start at least 15 years prior to expected symptom onset, with tau levels increasing 10 to 15 years before expected symptom onset (calculated as age of the participant minus parent’s age at symptom onset) [152, 153]. Despite differences in autosomal dominant and sporadic forms of AD—with the autosomal dominant variant associated with overproduction of Aβ42 in contrast to under clearance in the sporadic form [154, 155]—these findings are consistent with studies in the much more common sporadic form of AD [156–160]. The study by Fagan et al. [153], however, pointed to potential reductions in CSF tau once subjects had passed their age of expected symptom onset. Additional studies in sporadic AD have reported supportive findings [161, 162]. Possibly, this may reflect a deceleration in neuronal injury or variations in the number of neurons being affected at a given disease stage [153].

At the MCI stage of AD, longitudinal findings over the course of a nearly 10-year period have shown that CSF levels of Aβ42 were decreased 5 to 10 years prior to progression to AD dementia, whereas T-tau and P-tau appeared to be later markers as baseline levels were significantly higher in those who converted within the first 5 years, as compared to those who converted between five and years [100]. Though longitudinal studies with serial sampling over extended periods are lacking, longitudinal findings over shorter intervals (e.g., 4 years, with CSF sampled at three time points) have shown relative stability of CSF AD biomarkers [156, 163]. These studies also provided support for the hypothesis that tau follows Aβ pathology due the observation that low baseline Aβ42 values were associated with longitudinal increases in P-tau, but not the opposite. In line with this, Mattsson et al. recently showed that increases in CSF P-tau181 and P-tau217 appear to follow shortly after Amyloid PET [123].

### Phase 4: Prospective diagnostic studies

The general aim of phase 4 studies is to quantify the biomarker accuracy in patients diagnosed and treated based on biomarkers and perform preliminary assessment of usefulness in preparation of phase 5. Studies at this stage involve testing people and lead to diagnosis and treatment. Only preliminary evidence is available for the phase 4 aims.

#### Phase 4: Primary aim

To determine the operating characteristic of the biomarker in a representative population by determining the true and false positive referral rates leading toward diagnosis and treatment.

The primary aim of phase 4 is to determine the operating characteristics of the biomarker-based test in a real-world population by determining the detection and false referral rates. There is preliminary evidence for the phase 4 primary aim; longitudinal studies, however, are required for this aim to be fully achieved.

As described in the “Phase 3: Primary aim 2” section, a high (~90%) level of concordance has been reported between CSF Aβ42 and Amyloid PET [110]. In a study by Palmqvist et al., CSF Aβ42 and amyloid imaging using [^18^F]flutemetamol PET were compared in patients with MCI from the Swedish BioFINDER study [39]. CSF Aβ42, measured using consecutive samples as part of routine clinical practice by board-certified laboratory technicians at an accredited laboratory, showed high accuracy for determining cortical Aβ levels in MCI patients, as measured using [^18^F]flutemetamol PET, with 92% of patients identically classified. Similarly, in a study by Hansson *et al*., CSF T-tau/Aβ42 and P-tau/Aβ42 ratios showed a high level of agreement with Amyloid PET based classifications in BioFINDER (90% agreement and an AUC of 94%) and ADNI (89% agreement and an AUC of 96%) [26]. Remarkably, the ratios combing T-tau and P-tau with Aβ42 were shown to be as accurate as SUVR values in predicting Amyloid PET visual reads [26].

In the few studies that have addressed concordance between CSF tau and Tau PET [113, 122], overall concordance rates have varied between approximately 50% and 70% [113]. The overall lower concordance between tau biomarkers—as compared to Aβ [110]—may relate to Aβ biomarkers assuming a more bimodal distribution, as compared to tau measures [113]. The discrepant concordance findings between studies likely relate to differences in the cohorts studied (in terms of age, CSF tau levels and MMSE, for instance), interval between CSF sampling and PET, the use of different Tau PET tracers, and differences in the control subjects used to define Tau PET cut-points [122].

#### Phase 4: Secondary aim 1

To detect the predictive features of the biomarker, considering the potential benefits due to early detection. The first secondary aim of phase 4 secondary is partially achieved.

Early diagnosis of AD carries a number of advantages for patients and caregivers. These include optimized medical management, future planning, participation in clinical trials, risk reduction, and reduced overall care expenditures by delaying the transition to nursing home care [164–166]. There are also ethical concerns related to disclosing a diagnosis of AD at the MCI stage [166–168], mainly tied to the fact that there are as yet no treatments able to stop or modify the course of the disease. Despite this, CSF AD biomarkers are increasingly used in clinical practice in the evaluation of MCI patients, with a survey of European Alzheimer's Disease Consortium centers [169] showing that CSF AD biomarkers were reported to be used by 22% of responders, with 79% of respondents stating that they were very to extremely comfortable giving a diagnosis of MCI due to AD when all three markers were abnormal.

Studies examining the impact of CSF AD biomarkers on diagnosis and diagnostic confidence have shown these measures to be of value. Kester et al. [170] showed that knowledge of CSF profiles in a non-academic memory clinic changed the diagnosis in 10% of the cases and increased diagnostic confidence in one third of cases. In a follow-up study, CSF AD biomarkers were found be to be diagnostically helpful to clinicians in 75% of cases and led to a change in diagnosis in more than 50% of MCI patients [171]. In a study that examined all patients visiting a tertiary center for cognitive screening during a 1-year period [172], the use of CSF AD biomarkers led to a change in diagnosis in 7% of patients and a 5% increase in diagnostic confidence; CSF findings were also shown to affect clinical management (e.g., additional investigations, greater follow-up, and clinical trial selection) in 23% of patients. Similar findings were also recently described by Cognat et al. [173]. Other findings from a study that focused on the clinical utility of [^18^F]flutemetamol in a tertiary memory clinic setting [174], however, showed that the primary reason (57% of patients) for referral for Amyloid PET in MCI patients was a clinical suspicion of AD in the context of unclear or negative CSF findings. Furthermore, the addition of CSF Aβ42, P-tau and T-tau to demographic information, neuropsychological testing, and medial temporal lobe atrophy was found to improve the accuracy of the prognosis for progression to dementia over a 5-year period in MCI patients [175].

The clinical value of CSF AD biomarkers can also be assessed indirectly. Findings from a recent a large-scale (> 16000 patients) multicentric US study (Imaging Dementia—Evidence for Amyloid Scanning; IDEAS) [176] showed that knowledge of Amyloid PET status was associated with significant changes in diagnosis and patient management, including the use of drugs approved for the symptomatic treatment of AD, other relevant drugs addressing dementia risk factors, counseling (e.g., monitoring of medications, driving and home safety), and future planning (medical/financial decision making, advanced directives). Given the high concordance between CSF Aβ42/40 or Aβ42/P-tau (>90%) has with Amyloid PET [26], the clear benefits of testing for amyloid status shown by this study should also be relevant for CSF AD biomarkers.

#### Phase 4: Secondary aim 2

To assess the practical feasibility of implementing the biomarker-based diagnostic procedure and compliance of test-positive subjects with work-up recommendations. There is now preliminary evidence for the second secondary aim of phase 4.

Though assessing the practical feasibility of diagnostic programs and compliance of test-positive subjects with work-up and treatment recommendations may be of limited value in the absence of disease modifying treatments for AD, several studies indicate that the clinical use of CSF AD biomarkers is feasible. The Swedish Dementia Registry [177, 178]—a national quality registry on dementia disorders used by the majority (93%) of memory clinics in Sweden—has collected CSF AD biomarker data on a majority of patients [93, 179] and survey-based data also shows that CSF AD biomarkers are frequently used within European countries [180]. However, despite the low risk of complications [181–186], studies show that lumbar punctures (LPs) are often negatively viewed by older individuals in North America [187].

#### Phase 4: Secondary aim 3

The secondary aim 3 of phase 4 is to make preliminary assessments of the effects of biomarker testing on costs and burden associated with the disease. The third secondary aim of phase 4 is not achieved.

Several studies have addressed the potential economic impact of CSF AD biomarkers. Using a simulation model, Handels et al. [175] found that the use of CSF AD biomarkers in MCI patients resulted in an average gain in quality-adjusted life years of 0.046 and carried an average per patient cost of €432; this translated into an incremental cost-effectiveness ratio of €9,416. Other studies assessing the incremental cost-effectiveness of CSF AD biomarkers in a hypothetical scenario in which disease-modifying treatments are available also support CSF measures being cost-effective [188]. Similar findings have also been reported when looking at symptomatic treatments [189]. The prevalence of AD in a given population has also been shown to affect estimates of cost-effectiveness for CSF. Lee et al. [190] found that the diagnostic use of CSF AD biomarkers is only likely to be cost-saving if the prevalence of AD is greater than 15% following clinical assessment and standard MRI-based neuroimaging.

Few studies have assessed whether the use of CSF AD biomarkers results in lower mortality in AD. In a study by Bruandet et al. [191], it was found that in a cohort of cognitively impaired patients (AD, AD with cerebrovascular disease, and vascular dementia), survival was tied to the interval between initial symptoms and the first healthcare visit. As such, earlier diagnosis may reduce mortality. In patients with MCI due to AD, however, it is not known whether the use of CSF AD biomarkers in routine clinical practice would reduce mortality.

#### Phase 4: Secondary aim 4

The secondary aim 4 of phase 4 is to monitor disease occurring clinically but not detected by the biomarker testing protocol. The fourth secondary aim of phase 4 secondary is not achieved.

Approximately 5 to 8% of patients with AD according to both clinical and neuropathological criteria do not have a CSF profile consistent with AD [84, 192, 193]. As a result, the use of dichotomized CSF AD biomarkers to establish a diagnosis of AD would result in some false negatives. The extent of this problem, however, would also relate to the method used to set cut-offs defining what constitutes an abnormal value [189].

### Phase 5: Disease-control studies

Studies aiming to quantify the impact of CSF AD biomarker-based diagnosis in terms of reductions in disease-related morbidity/mortality, disability as well as the costs of biomarker testing in relation to patient costs (i.e., per life saved or quality-adjusted life year). This phase also aims to address patient compliance with screening and workup across varied settings and to compare different treatment approaches to biomarker-positive subjects and their effects on mortality and costs.

The primary aim of phase 5 is to test the capacity of a biomarker-based diagnosis to reduce the burden of AD. Secondary aims include examining patient compliance across different settings and comparing different protocols and associated benefits. As there are as yet no disease-modifying treatments for AD, phase 5 studies have not been performed; phase 5 is therefore not achieved.

## Discussion

In the present review, we aimed to update the previous work on validation status of CSF AD biomarkers [9], using a biomarker validation framework developed for oncology biomarkers [1]. Though the most important achievements, by comparison to the previous review on this topic, are the development and implementation of certified reference methods and materials for CSF Aβ42, the increasing use of fully automated assays for CSF AD biomarkers and a unified protocol for how CSF samples are to be handled (phase 2, secondary aim 1), advances in the level of evidence were also found for phases 3 (primary aim 2; secondary aims 1 to 3) and 4 (secondary aim 2).

In comparison to the previous Roadmap meeting, the first secondary aim of phase 2—dealing with the optimization of operating procedures and assay reproducibility—is now fully achieved*.* As outlined by Janelidze et al. [35], there now exists a protocol for the handling of CSF AD biomarkers. Together with the appropriate use criteria for LPs [33], this protocol could serve as the basis for a universal preanalytical protocol for CSF AD biomarkers, one that could be incorporated into routine AD diagnosis and future clinical trials [27]. The Alzheimer’s Associations is now leading its consensus-based approval by relevant stakeholders. The use of novel automated platforms will help provide CSF AD biomarker measurements that are both highly precise and stable; this, combined with CRMs, will facilitate the introduction of uniform cut-offs that can be applied across centers and laboratories, a key requirement for the routine use CSF AD biomarkers in memory clinics and in clinical trials with candidate disease-modifying drugs. The availability of CSF results that are both highly precise and stable across sample batches will also facilitate the pooling of CSF AD biomarker results across research centers, allowing for studies addressing the pathogenesis and progression of AD and related neurodegenerative disorders. Though a mass spectrometry-based method of quantification for T-tau has been developed [194], an important and as yet unmet prerequisite for the wider use of T-tau and P-tau measurements is the current lack of CRMs for assay standardization [47]. Advances similar to those for Aβ42 (i.e., the development and implementation of certified reference materials and methods) [22, 195] will hopefully soon follow for tau [196]**.**

In comparison to the previous Roadmap meeting, the second primary aim of phase 3—addressing the definition of criteria for biomarker positivity—is now fully achieved for CSF Aβ42 and partially achieved for P-tau and T-tau due the current lack of CRMs. Using the fully automated Elecsys® immunoassays, studies indicate a cut-off of 1100 pg/mL for CSF Aβ42 [26, 86, 87] and approximately 0.02 for P-tau/Aβ42 and 0.14 for T-tau/Aβ42 [26, 87, 88]. A cut-off of 0.068 has also been shown for Aβ42/Aβ40 using the LUMIPULSE® assay though additional studies are required to address ratios using Aβ42 and tau. With respect to the first secondary aim, which explores the impact of covariates on CSF AD biomarkers at the MCI stage, in agreement with findings from studies addressing the effects of age and *APOE* [9, 56, 60, 64, 90, 91], the Alzheimer’s Biomarkers Standardization Initiative concluded that there was no need to set different cutoffs for AD CSF biomarkers based on either of these variables [197], a position also articulated in the recent recommendations for the diagnostic use of these measures in the clinical work up of patients with MCI [198].

In addition to the primary and secondary aims of phase 3, secondary aims two and three are also now fully achieved. With respect to the second secondary aim, which aims to compare biomarkers, ratios combining Aβ42 with Aβ40, P-tau or T-tau have greater diagnostic utility compared to the use of individual CSF AD biomarkers. The superior performance of these ratios may be due to several reasons. Aβ42 in ratio with Aβ40 appears to compensate for between laboratory variations in the way CSF is processed [27, 199] and also for interindividual differences in Aβ production levels [200, 201]. The superiority of Aβ42 in ratio with either P-tau or T-tau may be due to the combination of two different pathological processes into one measure [26]. In addition, these ratios may account for natural differences in the production, secretion, and breakdown of CSF proteins [202]. By comparison to PET, CSF tau measures can be described primarily as markers of disease state, with Tau PET serving as a marker of disease stage. This position is supported by a recent study that used stable isotope labeling kinetics to monitor the half-life and turnover rate of tau in the human CNS [203] and by recent in vivo findings [123, 126]. Findings supportive of this model (i.e., that CSF and PET capture different aspects of AD pathology) have also been reported for Aβ-biomarkers [111, 112]. Lastly, based on studies addressing the third secondary aim, which aims to developed algorithms to combine CSF AD biomarkers with other measures, multicentric data supports the combined use of CSF AD biomarkers to predict progression from MCI to AD dementia at the individual patient level [150]. Though the findings of this study have yet to be prospectively evaluated, it is conceivable that the models developed as part of this study could be used in clinical practice [204].

For phase 4, preliminary evidence now supports the widespread use (feasibility) of CSF AD biomarkers. This achievement rating, however, is based on European studies. In North America, many older adults have a negative perception of LPs [187] despite very limited supportive evidence [181–186]. Moreover, while it has been shown that a majority of older Americans are willing to undergo a LP for medical reasons if useful information pertaining to their health can be gained [205], enthusiasm for an LP solely for research purposes was limited. Though this finding contradicts the commonly held belief that North Americans are unwilling to undergo LPs, the authors found no modifiable factors that could improve the perception of LPs among those who view the procedure negatively [205]. Some of the perceived difficulties in performing LPs in North America, however, may relate to clinician bias, care delivery models and low reimbursement rates for LPs [206, 207]. Further studies are required to explore these issues.

Several limitations apply to this review. First, although our approach adhered to a sound methodology, rating degree of achievement for each aim should be based on a more thorough assessment of evidence, including examining various possible sources of bias (e.g., GRADE guidelines) [20]. Our online material is meant to help this development as a next step forward in a systematic assessment of the validation of AD biomarkers. Third, in reviewing phase 3 studies, clinical diagnosis, as opposed to post-mortem diagnosis, was used as the SOT. Lastly, though the focus of this review was the performance of CSF AD biomarkers in MCI patients, the definition of MCI was not homogeneous across studies.

## Conclusions

We herein addressed the validation maturity of CSF Aβ42, P-tau, and T-tau for the diagnosis of AD at the MCI stage. Though much has been achieved for phases one through three, much work remains to complete phases four and five, dealing with the performance of CSF AD biomarkers in representative memory clinic cohorts and health care outcomes. To a large degree, this will depend on the availability of treatments capable of modifying or stopping the course of AD.

## Supplementary information

ESM 1 (DOCX 19 kb)
