# Supplementary material for: 2020 update on the clinical validity of cerebrospinal fluid amyloid, tau, and phospho-tau as biomarkers for Alzheimer’s disease in the context of a structured 5-phase development framework
Source: Eur J Nucl Med Mol Imaging. 2021 Mar 5;48(7):2121–39. doi: 10.1007/s00259-021-05258-7 (PMC8175301; doi:10.1007/s00259-021-05258-7)
Supplement: Supplementary file 1 — (DOCX 19 kb) [file 259_2021_5258_MOESM1_ESM.docx]

**Online Resource 1.** PubMed search strings

**Phase 1**

*As this phase was deemed to be fully achieved during the first Geneva Roadmap meeting, a literature search was not performed for this phase*

**Phase 2**

***Primary aim***

("accuracy" OR "sensitivity" OR "Specificity" OR "ROC" OR "predictive value") AND ("Alzheimer") AND ("Healthy Controls" OR "Cognitively normal" OR "controls" OR "normal") AND (*tracer) AND (other disease-if pertinent)

***Secondary aim 1***
("effect" OR "accociation" OR "covariates") AND ("factor" OR "habit" OR "age" OR "sex" OR "gender" OR "education" OR "life-style" OR "risk factor") AND ("*Alzheimer") OR ("healthy controls" OR "cognitively normal" OR "controls" OR "normal") OR ("MCI" OR "mild cognitive impairment" OR "prodromal") AND ("CSF" or "cerebrospinal") AND ("amyloid" OR "tau")
***Secondary aim 2***
("follow-up” OR “followup” OR “conversion” OR “progression” OR “decline” OR "predict" OR "cut-off" OR "cut-point" OR "measure" OR "assessment") AND ("combinat" OR "associat" OR "compar") AND (“Alzheimer”) AND ("MCI"OR "mild cognitive impairment" OR "prodromal") AND ("CSF" OR "cerebrospinal") AND ("amyloid" OR "tau")
***Secondary aim 3***
("follow-up” OR “followup” OR “conversion” OR “progression” OR “decline” OR "predict" OR "cut-off" OR "cut-point" OR "measure" OR "assessment") AND ("combinat" OR "associat" OR "compar") AND (“Alzheimer”) AND ("MCI"OR "mild cognitive impairment" OR "prodromal") AND ("CSF" OR "cerebrospinal") AND ("amyloid" OR "tau")

***Secondary aim 4***
("follow-up” OR “followup” OR “conversion” OR “progression” OR “decline” OR "predict" OR "cut-off" OR "cut-point" OR "measure" OR "assessment") AND ("combinat" OR "associat" OR "compar") AND (“Alzheimer”) AND ("MCI"OR "mild cognitive impairment" OR "prodromal") AND ("CSF" OR "cerebrospinal") AND ("amyloid" OR "tau")

**Phase 3**

***Primary aim***

("follow-up" OR "followup" OR "conversion" OR "progression" OR "decline" OR "predict") AND ("MCI" OR "mild cognitive impairment" OR "prodromal") AND ("CSF" OR "cerebrospinal") AND ("amyloid" OR "tau") AND ("cut-off" OR "cut-point" OR "measure" OR "assessment")

***Secondary aim*** ***1***
("effect" OR "accociation" OR "covariates") AND ("factor" OR "habit" OR "age" OR "sex" OR "gender" OR "education" OR "life-style" OR "risk factor") AND ("*Alzheimer") OR ("healthy controls" OR "cognitively normal" OR "controls" OR "normal") OR ("MCI" OR "mild cognitive impairment" OR "prodromal") AND ("CSF" or "cerebrospinal") AND ("amyloid" OR "tau")

***Secondary aim*** ***2***
("follow-up” OR “followup” OR “conversion” OR “progression” OR “decline” OR "predict" OR "cut-off" OR "cut-point" OR "measure" OR "assessment") AND ("combinat" OR "associat" OR "compar") AND (“Alzheimer”) AND ("MCI"OR "mild cognitive impairment" OR "prodromal") AND ("CSF" OR "cerebrospinal") AND ("amyloid" OR "tau")
***Secondary aim*** ***3***
("follow-up” OR “followup” OR “conversion” OR “progression” OR “decline” OR "predict" OR "cut-off" OR "cut-point" OR "measure" OR "assessment") AND ("combinat" OR "associat" OR "compar") AND (“Alzheimer”) AND ("MCI"OR "mild cognitive impairment" OR "prodromal") AND ("CSF" OR "cerebrospinal") AND ("amyloid" OR "tau")
***Secondary aim*** ***4***
("follow-up” OR “followup” OR “conversion” OR “progression” OR “decline” OR "predict" OR "cut-off" OR "cut-point" OR "measure" OR "assessment") AND ("combinat" OR "associat" OR "compar") AND (“Alzheimer”) AND ("MCI"OR "mild cognitive impairment" OR "prodromal") AND ("CSF" OR "cerebrospinal") AND ("amyloid" OR "tau")

**Phase 4**

***Primary aim 1***

("diagnosis" OR "treatment") AND ("Alzheimer") AND ("MCI" OR "mild cognitive impairment" OR"prodromal") AND ("CSF" OR "cerebrospinal") AND ("amyloid" OR "tau")

***Secondary aim 1***
(“clinical diagnosis” OR “treatment” OR “memory clinic”) AND (“benefits"OR “outcome” OR “improve”) AND (“Alzheimer”) AND ("MCI" OR "mild cognitive impairment" OR "prodromal") AND AND ("CSF" OR "cerebrospinal") AND ("amyloid" OR "tau")
***Secondary aim 2***
("clinical diagnosis” OR “treatment” OR “memory clinic”) AND ("benefit" OR "compliance" OR "mortality" OR"morbidity" OR "QoL" OR "quality of life") AND (“Alzheimer”) AND ("CSF" OR "cerebrospinal") AND ("amyloid" OR "tau")
***Secondary aim 3***
("clinical diagnosis” OR “treatment” OR “memory clinic”) AND ("benefit" OR "compliance" OR "mortality" OR"morbidity" OR "QoL" OR "quality of life") AND (“Alzheimer”) AND ("CSF" OR "cerebrospinal") AND ("amyloid" OR "tau")

***Secondary aim 4***
("clinical diagnosis” OR “memory clinic” OR “criteria” OR "recommendation") AND ("accuracy" OR "sensitivity" OR "specificity" OR"ROC" OR "predictive value" OR "concordance" OR "confirm" OR "negative detection rate" OR "negative referral rate" OR "false negative rate") AND (“Alzheimer”) AND ("CSF" OR "cerebrospinal") AND ("amyloid" OR "tau")

**Phase 5**

***Primary aim 1***

("diagnosis" OR "detection") AND ("benefit" OR "compliance" OR "mortality" OR "morbidity" OR "QoL" OR "quality of life" OR "financial impact" OR "cost" OR "effectiveness") AND ("Alzheimer") AND ("CSF" OR "cerebrospinal") AND ("amyloid" OR "tau")

***Secondary aim 1***
("diagnosis" OR "detection") AND ("benefit" OR "compliance" OR "mortality" OR "morbidity" OR "QoL" OR "quality of life" OR "financial impact" OR "cost" OR "effectiveness") AND ("Alzheimer") AND ("MCI" OR "mild cognitive impairment" OR "prodromal) AND ("CSF" OR "cerebrospinal") AND ("amyloid" OR "tau")
***Secondary aim 2***
("diagnosis" OR "detection") AND ("benefit" OR "compliance" OR "mortality" OR "morbidity" OR "QoL" OR "quality of life" OR "financial impact" OR "cost" OR "effectiveness") AND ("Alzheimer") AND ("CSF" OR "cerebrospinal") AND ("amyloid" OR "tau")
***Secondary aim 3***
* ("diagnosis" OR "treatment") AND ("protocol" OR "recommendation" OR "criteria") OR (AND ("benefit" OR "compliance" OR "mortality" OR "morbidity" OR "QoL" OR "quality of life") AND ("financial impact" OR "cost" OR "effectiveness") AND ("Alzheimer") AND ("CSF" OR "cerebrospinal") AND ("amyloid" OR "tau")
